# Reasons for mini-implants failure: choosing installation site should be valued!

**DOI:** 10.1590/2176-9451.19.2.018-024.oin

**Published:** 2014

**Authors:** Alberto Consolaro, Fábio Lourenço Romano

**Affiliations:** 1 Full professor, School of Dentistry - University of São Paulo/Bauru and professor at the postgraduate program at the School of Dentistry -University of São Paulo/ Ribeirão Preto; 2 PhD in Orthodontics, School of Dentistry, State University of Campinas/Piracicaba. Professor at the undergraduate and postgraduate programs, School of Dentistry -University of São Paulo/ Ribeirão Preto

**Keywords:** Mini-implants, Absolute anchorage, Micro-screws, Temporary anchorage devices

## Abstract

Mini-implant loss is often associated with physical and mechanical aspects that result from choosing an inappropriate placement site. It is worth highlighting that:

a) Interdental alveolar bone crests are flexible and deformable. For this reason, they may not offer the ideal absolute anchorage. The more cervical the structures, the more delicate they are, thus offering less physical support for mini-implant placement; b) Alveolar bone crests of triangular shape are more deformable, whereas those of rectangular shape are more flexible; c) The bases of the alveolar processes of the maxilla and the mandible are not flexible, for this reason, they are more likely to receive mini-implants; d) The more cervical a mini-implant is placed, the higher the risk of loss; the more apical a mini-implant is placed, the better its prognosis will be; e) 3D evaluations play a major role in planning the use of mini-implants.

Based on the aforementioned considerations, the hypotheses about mini-implant loss are as follows:

1) Deflection of maxillary and mandibular alveolar processes when mini-implants are more cervically placed; 2) Mini-implants placed too near the periodontal ligament, with normal intra-alveolar tooth movement; 3) Low bone density, low thickness and low alveolar bone volume; 4) Low alveolar cortical bone thickness; 5) Excessive pressure inducing trabecular bone microfracture; 6) Sites of higher anatomical weakness in the mandible and the maxilla; 7) Thicker gingival tissue not considered when choosing the mini-implant.

## Abstract (pt)

As perdas de mini-implantes estão quase sempre relacionadas aos aspectos físicos e mecânicos decorrentes de uma escolha inadequada do local de inserção. Deve se destacar que:

a) As cristas ósseas alveolares interdentárias têm flexão e se deformam, e podem não oferecer ancoragem tão absoluta. Quanto mais cervicais, as estruturas são mais delicadas e oferecem menos suporte físico para os mini-implantes; b) as cristas ósseas alveolares triangulares se deformam mais, e as retangulares são menos flexíveis; c) as bases do processo alveolar nos corpos da maxila e mandíbula não têm capacidade flexiva, e seu volume e estruturas são maiores, logo, são mais receptivas para mini-implantes; d) quanto mais próximo da cervical se coloca um mini-implante, maior é o risco de se perdê-lo; quanto mais apical se coloca o mini-implante, melhor é o seu prognóstico; e) avaliar a região tridimensionalmente representa um passo fundamental no planejamento do uso de mini-implantes.

Com base nessas considerações, as hipóteses para a perda de mini-implantes são:

1) Deflexão do processo alveolar da maxila e mandíbula, quando fixados em posições mais cervicais; 2) proximidade com o ligamento periodontal e o movimento dentário intra-alveolar normal; 3) densidade óssea menor, pouca espessura e menor volume ósseo alveolar; 4) espessura menor da cortical óssea alveolar; 5) pressão excessiva, induzindo microfraturas ósseas trabeculares; 6) locais de maior fragilidade anatômica mandibular e maxilar; 7) espessura maior do tecido gengival não considerada na escolha do mini-implante.

## Introduction: Mechanical interlocking is what matters!

Dental implants conceptually required months for crown placement and functional recovery. It was believed that it took weeks or months for cells to colonize the implant surface, produce matrix and mineralize it by efficient osseointegration. Science developed and now it is possible to apply a functional load nearly immediately after implants are placed, provided that they are mechanically interlocked in a previously mineralized bone. Appropriate mechanical interlocking between implant and bone surfaces allow mini-implants to withstand masticatory forces while gradual osseointegration occurs. The type of bone and clinical condition will determine whether immediate-load implants are recommended or not.

Absolute anchorage during orthodontic treatment may be obtained with mini-implants^2^ or miniplates^9^ placed in thick cortical bone and dense trabecular bone. Mini-implant threads must be perfectly fitted or adapted to the bone where they are inserted, allowing stability and nearly immediately withstanding the forces applied. Mini-implants are also known as micro-implants, micro-screws or anchorage screws, and comprise what is known as temporary anchorage devices (DAT).

Some specialists suggest that forces may be applied 3 days after mini-implant placement, while others recommend a waiting period of 21 or 40 days. As for immediate loading for absolute anchorage mini-implants, the waiting time is shorter - in fact, it could be immediately applied.^3,12^

Cortical bone thickness and trabecular bone density are important factors to be considered when determining a mini-implant placement site. Moreover, the material, surgical technique, patient's hygiene care and patient control performed by the professional are also of paramount importance.^7^

The main mechanism related to the efficiency of mini-implants for absolute anchorage in Orthodontics is their mechanical adaptation to previously mineralized bone structures. Such mechanism is also known as interlocking. After a few weeks or months, osseointegration, represented by cell colonization and bone formation on the surface of mini-implants, will be of secondary importance, given that, at this point, mini-implants can be removed after fulfilling their role of orthodontic anchorage.

Osseointegration is the essence of Implantodontics and is responsible for the success in the esthetic and functional recovery of lost teeth. For this reason, mechanical adaptation allows the application of immediate load within the first weeks/months. In Orthodontics, on the other hand, mechanical adaptation explains the use of mini-implants for absolute anchorage, while osseointegration is secondary and belated. Orthodontic treatment with mini-plants lasts 30% less. Furthermore, the use of such devices allow orthodontic movement to be performed without further side effects on other teeth.^2^

## Characteristics of mini-implants and common consequences

Osseointegration may hinder mini-implant removal and increase the risk of fracture. For this reason, mini-implants are made of titanium metal alloy, pure grade V. In 2007, Vannet et al^11^ placed mini-implants in dogs and histomorphometrically determined that partial osseointegration occurred in all specimens 6 months after skeletal anchorage. Mini-implants placed in thinner bone and cortical bones may require osseointegration. In these cases, titanium alloy pure grade IV is used, with acid attack on the surface of mini-implants to increase contact surface.

Thread shape and length are essential for mini-implant placement. Resistance to fracture may be improved with cone-shaped mini-implants and perforating threads. These characteristics aid dissipation of compression forces exerted by bone structures surrounding the mini-implant while it is being installed.

**Figure 1:**
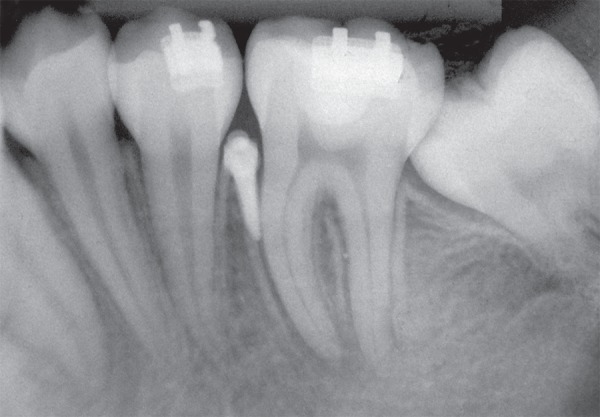
Mini-implant near the dental root.

**Figure 2:**
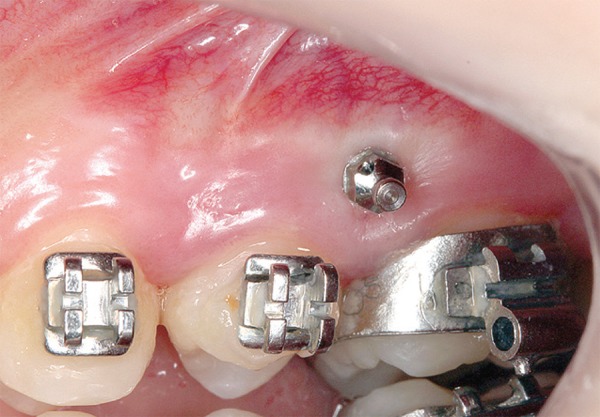
Mucositis around a mini-implant.

**Figure 3:**
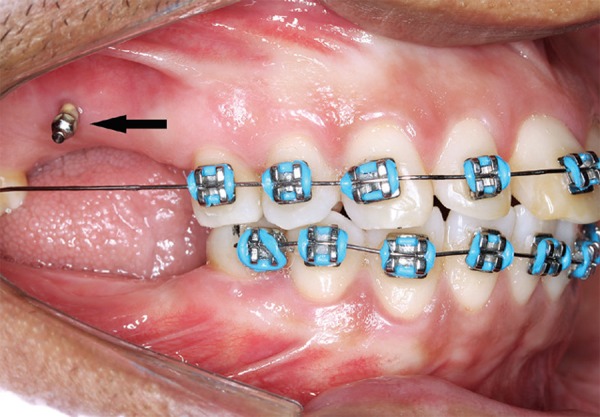
Food debris around a mini-implant.

**Figure 4:**
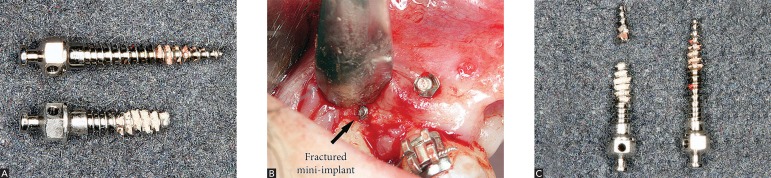
Mini-implant fracture during placement.

Mini-implant placement is simple, provided that it is carried out by skillful hands and prepared minds. On the other hand, it may offer risks when mistakenly planned and performed. According to Kyung et al^6^ and Reynders et al,^7,8^ mini-implant success depends on the surgeon's ability, patient's condition, appropriate placement site, initial stability, orthodontic mechanics, type of mini-implant and oral hygiene. The most frequent complications and accidents are contact between adjacent tooth roots (Fig 1), mucositis (Fig 2), contamination (Fig 3) and mini-implant fracture during placement (Fig 4) or removal. Other authors highlight that inflammation of soft tissues surrounding the mini-implant is a potential complication for TADs, which also contributes to loss of stability.^4,5,10^

**Figure 5:**
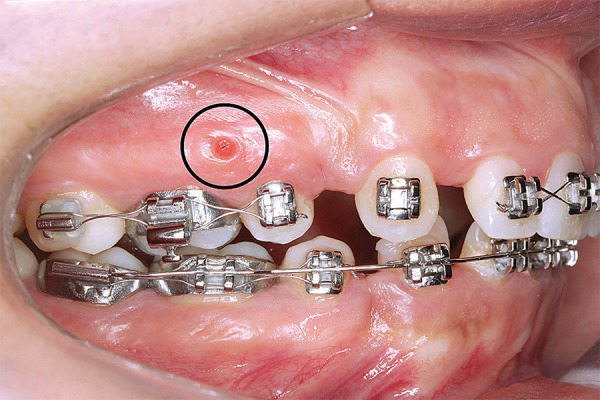
Mini-implant loss.

However, one of the most frustrating consequences is mini-implant loss during absolute anchorage, when the mini-implant is dislocated and unscrewed (Fig 5). Many hypotheses try to explain the 20% rate of mini-implant loss during orthodontic treatment. This paper aims at discussing the most reasonable theories by expanding the biological and clinical knowledge gathered within Implantodontics and adapting the concepts to mini-implants and orthodontic absolute anchorage.

## Hypotheses that explain mini-implant loss during orthodontic absolute anchorage

### 1) Deflection of maxillary and mandibular alveolar processes and mini-implant displacement: the more apical a mini-implant is placed, the better!

The alveolar process is the portion of maxillary and mandibular bone in which teeth are suspended. It is of relatively fragile buccal and lingual thickness, with structures in continuity with the main part of the maxilla and mandible. In an oral context, it is mostly comprised, in terms of volume, by tooth roots.

During orthodontic movement, a portion of the forces applied to the teeth promotes alveolar bone deflection. Likewise, such deflection should also occur during mini-implant anchorage, which hinders or interrupts the mechanical interlocking necessary between a mini-implant and the bone, thus, resulting in mini-implant displacement and loss.

**Figure 6:**
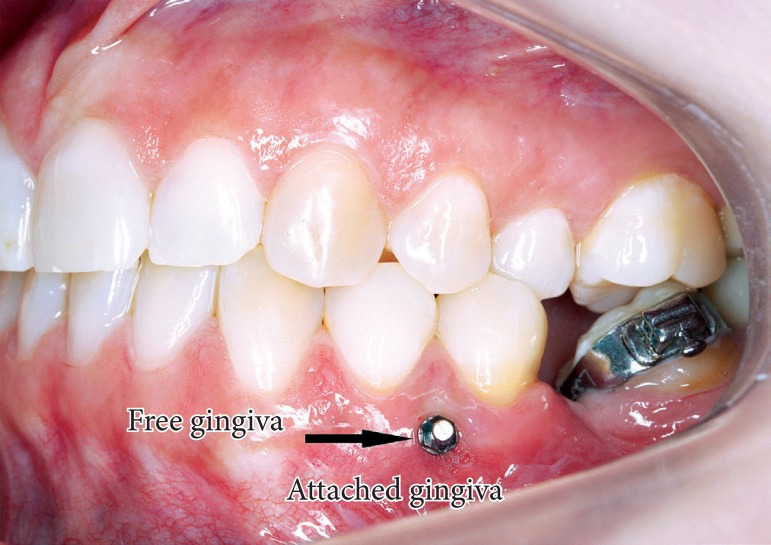
Appropriate mini-implant placement.

To avoid mini-implant loss as a result of alveolar bone deflection, the device must be placed as near the alveolar process base as possible. In other words, in the apical third of the roots where bone volume, cortical thickness as well as thicker and denser trabecular bone prevent any structural movement from happening as a result of bone deflection. Nevertheless, clinically speaking, this is not always possible, given that a mini-implant must be preferably placed in the attached gingiva in order to offer greater comfort to patients (Fig 6).^2^

### 2) Mini-implants placed too near the periodontal ligament with normal intra-alveolar tooth movement: Movement leads to structural lesions and inflammation!

The periodontal ligament is a specialized connective tissue fiber with 50% of its volume comprised by blood vessels. It is, on average, 0.25 mm thick. A tooth constantly leaves and enters into the socket during mastication, occlusion, swallowing, among other functions. Such intra-alveolar movements are softened and limited by periodontal collagenous and elastic fibers.

**Figure 7:**
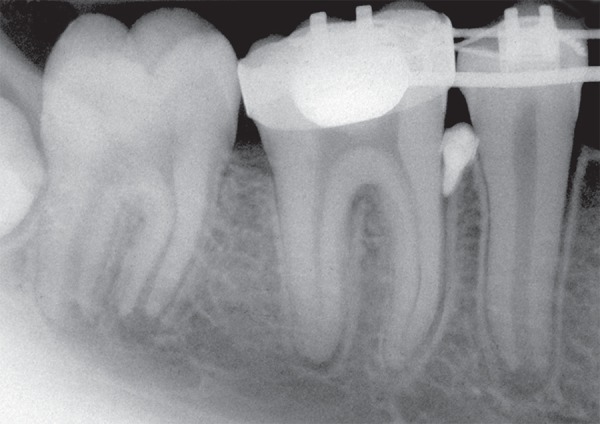
Mini-implant placed in the periodontal ligament.

When a mini-implant is placed too near the periodontal ligament, it causes friction between a movable piece - the tooth - and a fixed piece (Fig 7), which not only causes blood vessels, cells and fibers to break, but also stimulates inflammation and, as a consequence, peri-implant bone resorption and mechanical interlocking loss.

A mini-implant must not directly touch or be placed too near a tooth due to tooth movement. Considering mini-implant position and composition, the device is not harmful to tissues. When mini-implants are placed between teeth, they must be at least 1 mm away from the roots on both sides.

### 3) Low bone density, thickness and alveolar bone volume

Mechanical interlocking, essential for a mini-implant to provide absolute anchorage, requires consistent bone structure with thick cortical plate and dense cancellous bone with thick and numerous trabeculae. In the alveolar processes, the more cervical, the thinner the cortical plates and trabeculae tend to be.

**Figure 8:**
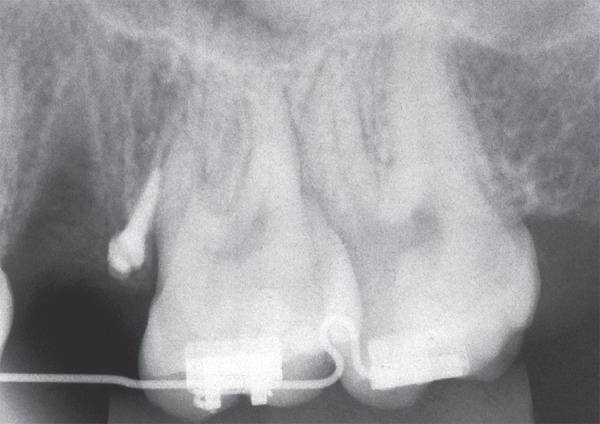
Mini-implant placed in inappropriate alveolar bone.

Determining the optimal site for mini-implant placement is key to success in absolute anchorage. The buccal/lingual bone structure of the alveolar process is fragile and thin. The trabecular bone may be deeply extended between roots, but its fragility remains. In short, the more apical a mini-implant is placed, the more resistant structures are available, with denser and more voluminous cortical plates and cancellous bone. Mini-implants placed near sites of recent tooth extraction represent technical difficulties and potential risks of implant loss due to low volume and low amount of bone (Fig 8).

### 4) Low alveolar cortical bone thickness

**Figure 9:**
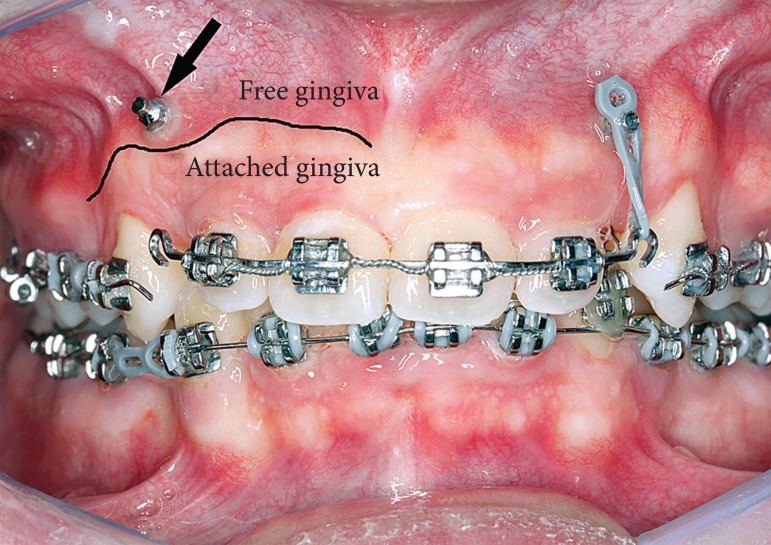
Mini-implant placed in free gingiva.

The alveolar cortical bones in the upper part of the maxilla and lower part of the mandible are much thicker. The different layers of cortical bone associated with the trabeculae from the cortical plates offer physical support as a result of interlocking with mini-implants. The closer the cortical plates are to the alveolar bone crests, the thinner they are, with the area over the teeth having the potential to present dehiscence or fenestration. It is worth reaffirming that the more apically a mini-implant is placed, the more successful absolute anchorage will be. However, we should always bear in mind that mini-implants placed on free gingiva may cause inflammation or edema as a result of tissue movement (Fig 9).

### 5) Excessive pressure and trabecular bone micro-fractures

Although mini-implants may be placed with relatively standardized forces, they may undergo overload due to excessive pressure applied by the operator during the procedure. Excess forces at mini-implant mechanical interlocking with underlying and peri-implant bone structures may lead to trabecular microfractures, peripheral and imperceptible micro-hemorrhage, and necrosis caused by the death of osteoblasts and osteocytes. Without these cells, the trabeculae and cortical bone tend to be reabsorbed by inflammation established around a mini-implant, which may result in mini-implant loss. To avoid excess pressure on alveolar bone during mini-implant placement, the specialist must apply gentle pressure so as to promote the initial interlocking. Subsequently, he rotates the wrench following the direction of the thread until the mini-implant platform touches the gingival tissue.

### 6) Sites of higher anatomical weakness in the mandible and the maxilla

The maxilla and the mandible consist of several muscles and tendons. They also hold teeth and soft tissues associated with the functions of the head and neck. Maxillary bones undergo inflammatory and reactive processes associated with periodontal disease, tooth eruption, bruxism, mastication, etc.

The anatomy of the maxilla and mandible comprise different thickness, density, volume and structures. Human maxilla and mandible vary considerably in volume, density and organization of bone structures as a result of adaptation to the specific conditions of each individual.

In the retromolar trigone, for instance, the triangular shaped area formed by two thick cortical plates located to the distal face of the second or third molar tends to be a spongeous, little dense bone unable to support absolute anchorage. Should mini-implants need to be placed at this site, they must be installed at their most lingual/buccal portions, which correspond to cortical plates that are thick in width and length.

**Figure 10:**
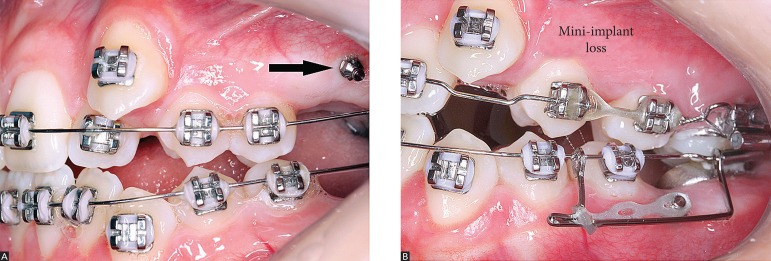
Mini-implant loss due to placement in recent extraction site.

The anatomical shape of the placement site must be carefully analyzed, especially tridimensionally. In the cavity between the lateral incisor and the canine, bone density and cortical thickness tend to be lower, similarly to recent extraction sites. The alveolar density and cortical plates of recent extraction sites are under functional remodeling, which hinders mini-implant placement in these areas (Fig 10).

### 7) Thicker gingival tissue not considered when choosing the mini-implant

Gingival soft tissue thickness must be considered when choosing the most appropriate type of mini-implant. In cases of thicker gingival tissues, the extraosseous part of a mini-implant represents the moment arm. This requires that a larger portion of the mini-implant be deeply inserted into the underlying bone structure so as to counterbalance the extraosseous moment arm. Should this factor not be considered when choosing the mini-implant design, mini-implant loss may occur as a result of movements of the implanted bone area promoted by absolute anchorage. In these areas, transmucosal profile (2 to 4 mm) and mini-implant length must be greater.

## Are oral bacteria, anesthesia and previous lancing procedures able to explain mini-implant loss?

Bacteria that reach tissues and mini-implants during placement are isolated. They are in low numbers and, therefore, not enough to trigger an inflammatory process that is worse than inflammation resulting from surgical procedures. Bacteria isolated from microbial biofilms are easily controlled by phagocytosis and destroyed by inflammatory exudate and infiltrate. Alone, they are not able to trigger inflammatory processes or consequential bone resorption that could lead to mini-implant loss. These bacteria are the same that cause periodontal disease; however, they do not promote mucositis or peri-implantitis in conventional implants either, provided that they do not form microbial biofilms. In 2013, Andruciolli^1^ conducted an *in vivo *study to assess microbial contamination by using DNA probes for 40 species of bacteria and the molecular biology technique of Checkerboard DNA-DNA hybridization. Bacterial endotoxin and inflammatory cytokines found in lost mini-implants were also used. The author concluded that microbial contamination and the amount of endotoxin found in the mini-implants did not act as a determining factor for loss of stability.

## Final considerations: Mini-implant loss is associated with the placement site!

The biology of cells and bacteria do not explain mini-implant loss. Our cells as well as our immune system readily accept titanium alloys, as reported by many researches. As for bacteria, they are the same of our microbiota. Thus, when they reach a tissue, they are soon defeated, as microbiota bacteria are. Mini-implant loss is often associated with physical and mechanical aspects that result from choosing an inappropriate placement site.

It is worth highlighting that:

1. Interdental alveolar bone crests are flexible and deformable. For this reason, they have little mobility to offer and may not provide the ideal absolute anchorage. The more cervical the structures, the more delicate they are, thus offering less mechanical interlocking for mini-implant placement.
2. Alveolar bone crests of triangular shape are more deformable, whereas those of rectangular shape are more flexible.
3. The bases of alveolar processes of the maxilla and the mandible are not flexible, for this reason, they are more likely to receive mini-implants.
4. The more cervical a mini-implant is placed, the higher the risk of loss. The more apical a mini-implant is placed, the better its prognosis will be.
5. Before mini-implant placement, it is advisable that a 3D analysis be carried out on the site by means of periapical radiographs, particularly by bisection and interproximal techniques, and occlusal radiograph with periapical film. Volumetric computed tomography with its several evaluation slices may replace conventional radiography.
